# Non-lesional mesial temporal lobe epilepsy requires bilateral invasive evaluation

**DOI:** 10.1016/j.ebr.2021.100441

**Published:** 2021-03-27

**Authors:** Ghazala Perven, Irina Podkorytova, Kan Ding, Mark Agostini, Sasha Alick, Rohit Das, Hina Dave, Marisara Dieppa, Alexander Doyle, Jay Harvey, Bradley Lega, Rodrigo Zepeda, Ryan Hays

**Affiliations:** a Department of Neurology, University of Texas Southwestern Medical Center, 5323 Harry Hines Blvd, Dallas, TX 75390-8508, USA; b Department of Neurosurgery, University of Texas Southwestern Medical Center, 5323 Harry Hines Blvd, Dallas, TX 75390-8855, USA

**Keywords:** Temporal lobe epilepsy, Normal MRI, Stereoelectroencephalography, MTLE, mesial temporal lobe epilepsy, SEEG, stereoelectroencephalography, RNS, responsive neurostimulator system

## Abstract

**Purpose:**

Mesial temporal lobe epilepsy (MTLE) usually responds well to surgical treatment, although in non-lesional cases up to 50% of patients experience seizure relapse. The possibility of bilateral independent seizure onset should be considered as a reason for epilepsy surgery failure.

**Methods:**

In a cohort of 177 patients who underwent invasive presurgical evaluation with stereo-tactically placed electrodes in two level four epilepsy centers, 29 had non-lesional MTLE. Invasive evaluation results are described.

**Results:**

Among 29 patients with non-lesional MRI and mesial temporal lobe seizure onset recorded during stereo-EEG (SEEG) evaluation, four patients with unilateral preimplantation hypothesis had independent bilateral mesial temporal seizures on SEEG despite of unilateral non-invasive evaluation data. Three of these patients were treated with bitemporal responsive neurostimulator system (RNS). Independent bilateral mesial temporal seizures have been confirmed on RNS ECoG (electrocorticography). The fourth patient underwent right anterior temporal lobectomy.

**Conclusion:**

We propose that patients with non-lesional mesial temporal lobe epilepsy would benefit from bilateral invasive evaluation of mesial temporal structures to predict those patients who would be at most risk for surgical failure. Neurostimulaiton could be an initial treatment option for patients with independent bitemporal seizure onset.

## Highlights

• Bilateral ictal onsets may lead to surgery failure in mesial temporal lobe epilepsy.
• Bitemporal SEEG seizures were recorded despite of unilateral non-invasive tests.
• Patients with non-lesional MTLE need bitemporal invasive evaluation before resection.

## 1 Introduction

Mesial temporal lobe epilepsy (MTLE) is the most frequent type of epilepsy that is referred to the epilepsy surgical centers, representing between 50 and 76% of all cases assessed for epilepsy surgery [1]. It usually responds well to surgical treatment, although in non-lesional cases up to 50% of patients experience seizure relapse after an anterior temporal lobe resection [2], [3]. One possibility of these surgical failures could be bilateral independent mesial temporal seizure onset missed inadvertently during pre-surgical evaluation. For instance, seizure onset from the temporal lobe contralateral to subdural grid (SDG) placement was described in patients with seizure relapse after the standard anterior temporal lobectomy [4]. Responsive Neurostimulation (RNS, manufacturer - Neuropace, Mountain View, California USA) studies reporting chronic implantation with bilateral mesial temporal lobe electrodes in patients with drug-resistant focal seizures have confirmed bilateral mesial temporal seizure origin in patients who had unilateral temporal seizures recorded with scalp EEG, but these patients were suspected to have bilateral mesial temporal epileptogenic zones based on the other standard localization testing [5], [6].

To our knowledge, no study has ever observed independent bilateral mesial temporal ictal onset on the stereo-EEG (SEEG) recording in non-lesional temporal lobe epilepsy (TLE) patients with unilateral pre-surgical non-invasive evaluation data, and further confirmed based on chronic indwelling ECOG (electrocorticography) with RNS.

## 2 Methods

This is a retrospective, observational study. A total of 177 patients with drug-resistant focal epilepsy who underwent invasive pre-surgical evaluation with stereo-electrodes at UT Southwestern Medical Center and Parkland Memorial Hospital from October 2014 to March 2020 were analyzed. Among them, 29 had MTLE based on stereo-EEG (SEEG) evaluation results, and non-lesional brain MRI. We describe the pre-surgical (Phase I) and invasive video-EEG (Phase II) evaluation results in these patients. The pre-surgical evaluation results, SEEG implantation maps, and then SEEG monitoring findings were discussed by a multidisciplinary committee included at least three board-certified epileptologists, a neurosurgeon, a neuroradiologist and the neuropsychologists. The study was approved by the institutional review board.

## 3 Results

Among 29 patients with non-lesional MRI and mesial temporal lobe ictal onsets recorded during stereo-EEG (SEEG) evaluation, only one patient had independent bilateral temporal seizures captured by previous scalp video-EEG monitoring. However, 5 out of 28 patients with unilateral temporal scalp EEG seizures had bilateral independent mesial temporal SEEG seizures, although all non-invasive evaluation data suggested a unilateral temporal lobe epileptogenic zone in 4 out of 5 patients. One of these five patients had independent bilateral temporal scalp EEG epileptiform discharges suggesting possibility of bilateral temporal epileptogenicity and therefore was excluded from the further analysis. The pre-implantation hypothesis was based on seizure semiology, ictal and inter ictal scalp EEG findings in all four patients, a pre-operative neuropsychological evaluation (in three out of four patients), and magnetoencephalography (MEG) results (in one patient). Brain MRI and FDG-PET were normal in these four patients. Language lateralization was performed using Wada test (one patient) or fMRI (two patients, currently preferable language lateralization technique [7], [8]), and was based on handedness only in one patient (his health insurance denied fMRI and Wada test). The SEEG evaluation was recommended based on presence of non-lesional MRI in patients with presumed TLE to exclude the TLE mimics as a reason of potential resective surgery failure [9], [10], [11]. Also, according to our institutional SEEG evaluation protocol, all patients with a pre-implantation hypothesis suggesting temporal lobe epilepsy undergo bilateral sampling of the mesial temporal structures with stereo-electrodes [12]. Within described cohort, 4/28 (14%) patients had bilateral independent mesial temporal lobe seizures recorded during SEEG video-monitoring - despite the fact that non-invasive pre-surgical data suggested a unilateral hypothesis. Table 1 summarizes the pre-surgical evaluation results for each case.

**Table 1:** Pre-surgical evaluation results.

	**Patient 1**	**Patient 2**	**Patient 3**	**Patient 4**
**Gender/Age at epilepsy onset,y/Age at SEEG,y/Handedness**	F/26/30/R	F/36/39/R	M/33/39/R	M/32/44/R
**Lateralizing signs, semiology**	Ictal and post-ictal aphasia	Right RINCH	Versive head turn to the right before FTC	Versive head turn to the left before FTC
**Ictal EEG**	Left temporal	Left temporal, or non-lateralizing and non-localizing with left temporal evolution	Left temporal	Right temporal
**Interictal EEG**	SW Left temporal	SW Left temporal, Left TIRDA	SW Left temporal	SW Right temporal, IS Right temporal
**PET**	Normal	Normal	Normal	Normal
**MEG**	Not done	Left mid and lateral anterior temporal	Not done	Not done
**Neuropsychological evaluation**	Dominant frontotemporal dysfunction	Mostly normal, minor inefficiencies of dominant frontal lobe systems	Not done	Non-dominant mesial temporal dysfunction
**Wada/IAP**	Language left dominant	Not done	Not done	Not done
**fMRI**	Not done	Language left dominant	Not done	Language left dominant

FTC, focal to bilateral tonic-clonic seizure; IAP, intracarotid amobarbital procedure; IS, intermittent slow; RINCH, rhythmic ictal nonclonic hand motions; SW, sharp waves; TIRDA, temporal intermittent rhythmic delta activity.

The first three patients were deemed to be poor candidates for surgical resection based on their SEEG data, and therefore each was treated with bitemporal responsive neurostimulation. Independent bilateral mesial temporal lobe seizures with more active left (dominant) temporal lobe have been noted on RNS ECoG in all three patients during longitudinal follow-up. The fourth patient had clinical seizures from the non-dominant (right) temporal lobe, but also three short 11–15 second duration sub-clinical seizures from the opposite hippocampus while off anti-seizure medication during the SEEG evaluation; therefore, a standard right anterior temporal lobectomy was recommended, and the patient is currently 8.5 months seizure-free after resection on anti-seizure medications. Table 2 demonstrates scalp EEG, SEEG, RNS ECoG characteristics and seizure outcome.

**Table 2:** Scalp EEG, SEEG, RNS ECoG characteristics and seizure outcome.

	**Patient 1**	**Patient 2**	**Patient 3**	**Patient 4**
**Scalp EEG monitoring duration, days**	5	4	6	4
**Scalp EEG interictals**	Left temporal	Left temporal	Left temporal	Right temporal
**Scalp EEG ictal onsets (seizure type/number recorded)**	Left temporal (Aura of deja vu/1, FIA/6)	Left temporal (FIA/5)	Left temporal (SCS/1, FTC/2), Non-lateralizing (SCS/2), No EEG changes (aura, left tongue sensory/3)	Right temporal (FTC/3)
**SEEG monitoring duration/First contralateral seizure captured, days**	12/5	16/10	8/3	9/2
**SEEG interictals, Left hippocampus/Right hippocampus, %**	100/0	90/10	50/50	50/50
**SEEG ictal onsets (seizure type/number recorded)**	Left perirhinal cortex (FIA/10), Right hippocampus (Autonomic aura/1)	Left hippocampus (FIA/4), Right hippocampus (Autonomic aura/1)	Left hippocampus (FIA/1, SCS/8), Right hippocampus (FIA/3, SCS/1)	Right hippocampus (FTC/2, Aura of deja vu/4, SCS/1), Left hippocampus (SCS/3)
**RNS monitoring duration, months**	55	31	30	N/A
**RNS interictals, Left hippocampus/Right hippocampus, %**	99/1	50/50	50/50	N/A
**RNS ictal onsets, Left hippocampus / Right hippocampus**	62/40	91/9	122/38	N/A
**Surgery / FIA seizure frequency pre-op / seizure type and frequency 3 months before last follow up visit**	RNS/ 1 per week/ 0–2 per month, aura	RNS/ 2–3 per week/ 3–6 per month, FIA	RNS/ 1–2 per week/ 0–3 per month, FIA	Right temporal lobectomy/ 1–4 per month/ None
**Seizure outcome, ILAE / follow up duration, months**	II / 55	IV / 31	IV / 30	I / 8.5

FIA, focal impaired awareness seizure; FTC, focal to bilateral tonic-clonic seizure; ILAE, International League Against Epilepsy; RNS, responsive neuro stimulator; SCS, sub-clinical seizure.

## 4 Discussion

Identifying patients with bilateral mesial temporal onsets is critical for surgical decision-making and overall prognosis of seizure freedom. Seizure relapse from the temporal lobe contralateral to the side of surgery estimated to represent 12–30% of patients who failed surgery [10].

Misdiagnosis of bilateral MTLE as unilateral and subsequent temporal lobe resection may lead not only to seizure persistence after the temporal lobectomy, but also to neuropsychological worsening resulting in a decline in quality of life for patients not in seizure remission following surgery [13].

There are few studies describing invasive EEG ictal findings contralateral to pre-implantation hypothesis. A study by Smart et al. reported a patient with one clear left-sided temporal onset seizure and four left temporal maximum but more ambiguous seizure onsets recorded during scalp EEG evaluation. Later there were three right mesial temporal lobe seizures recorded during invasive EEG monitoring. This patient had dysmorphic changes in the right temporal lobe, but normal hippocampal architecture without sclerosis, and her FDG-PET revealed left mesial temporal lobe hypometabolism [14].

A prospective study by Vadera and colleagues reporting SEEG evaluations after SDG demonstrated one patient who had the epileptogenic zone localized with SEEG contralaterally to a previous temporal lobectomy, but his pre-SEEG hypothesis was bilateral [4]. Interestingly, the initial pre-SDG hypothesis was unilateral left temporal so the patient underwent unilateral left temporal invasive evaluation with SDG. Since the unilateral left mesial temporal ictal activity was captured, a left temporal lobectomy was performed, but the patient continued having seizures and eventually the ictal activity arising from the right temporal lobe was captured during the subsequent SEEG evaluation.

In our patient cohort, all four patients had normal brain MRI and symmetric PDG-PET metabolism. Two patients had left temporal ictal onset, one patient had right temporal ictal onset, and one patient had left temporal, as well as non-lateralizing and non-localizing seizures with best evolution over the left temporal region recorded during scalp-EEG evaluation, therefore our study demonstrates that mesial temporal lobe epilepsy, especially non-lesional, could be bilateral disease even if pre-surgical evaluations are consistent with a unilateral temporal lobe epilepsy hypothesis.

The fact that nonlesional patients may have bilateral independent seizure foci despite unilateral findings on scalp EEG have been demonstrated before [15], but in our patient cohort not only the ictal scalp EEG, but also all the other variables (semiology in all patients, and MEG and Neuropsychometrics if available) were lateralizing to one hemisphere, therefore our findings reinforce need in bilateral invasive implantation in patients with unilateral scalp EEG ictal findings. Questionable abnormalities outside of the presumed zone of seizure onset in the unilateral frontal lobe were noted in Patients 1 and 2 during the neuropsychological evaluation, but those abnormalities were attributed to “stress”. The neuropsychological findings of Patient 2 were described as “mostly normal cognitive profile”, and in Patient 1 as “essentially all performances were average or above, with the exception on verbal fluency and confrontation naming tasks, which could be affected by Topiramate and patient’s anxiety regarding the possibility of verbal deficit. While not a deficit, complex attention and executive functioning were somewhat inefficient – this could be due to the patient in the next room who kept screaming”.

The seizure phenotypes were not identical in our sample. Two patients were females who had seizures with impaired awareness arising from the left mesial temporal structures and autonomic auras (heat sensation or nausea) arising from the right mesial temporal structures which were not recognized as seizures before SEEG evaluation. Later during RNS ECoG analysis the patients were asked to document these sensations, and ictal ECoG correlates were seen when autonomic auras were reported. The third patient was a male with left temporal or non-localizing onset seizures followed by left temporal evolution recorded during scalp-EEG evaluation, he had both clinical and subclinical seizures recorded from right and left mesial temporal structures independently. The fourth (male) patient had seizures with impaired awareness started from the right hippocampus, and subclinical seizures arising from the left hippocampus during SEEG study.

In our patient cohort an average SEEG EMU stay (Table 2) was 11.5 days (range 8–16), and an average SEEG duration to express bitemporal seizures was 5 days (range 2–10), what is shorter than reported in RNS study [5] perhaps due to SEEG monitoring off anti-seizure medications.

According to current pre-surgical evaluation guidelines, MRI and FDG-PET non-lesional TLE is an indication for invasive evaluation to localize the seizure onset zone(s) [9]. An option to replace invasive EEG by RNS in selected patients to record longer durations to record seizures and also provide treatment at the same time was discussed at our surgical conferences. At our center, we feel SEEG evaluation is necessary to localize the epileptogenic zone(s) including possible bilateral mesial temporal ictal onsets and epileptogenic foci outside of mesial temporal lobe(s) (temporal neocortical, temporal lobe epilepsy mimics and “temporal plus” epilepsy) to guide the RNS leads placement because RNS sampling is very limited. Our MTLE SEEG evaluation strategy is to sample mesial temporal structures bilaterally in all patients with a pre-implantation hypothesis suggesting MTLE in addition to limbic network exploration [12]; but, there is not a consensus in the literature that suggests an optimal sampling strategy in this patient population.

Optimal balance of invasive diagnostic and therapeutic procedures is crucial for epilepsy surgery success and quality of life improvement of epilepsy patients. In our patient cohort, two surgeries that penetrate the brain were performed in 3 out of 4 patients but only 1 out of 4 underwent resective surgery exposing the majority to added risk of intractanial EEG. Selecting pre-surgical evaluation strategy and subsequent therapeutic epilepsy surgery, such factors as complications rate, timely review, and cost must be considered to optimize surgical decision making, cost-effectiveness and ultimately to improve quality of life of patients with drug-resistant epilepsy.

## 5 Conclusions

Our study suggests that a significant minority − 4/28 (14%) - of patients in our sample who had non-lesional mesial temporal epilepsy and a unilateral pre-implantation hypothesis were ultimately determined to have independent bitemporal seizures despite a unilateral non-invasive evaluation. Therefore, in our case series of MRI-PET-negative suspected unilateral TLE patients, we found that these patients could benefit from bilateral invasive evaluation of mesial temporal structures with stereo-electrodes to predict those patients who would be at most risk for surgical failure and neuropsychological worsening. We speculate that responsive neurostimulation could be an initial diagnostic and treatment option for selected patients with independent bitemporal seizure onset followed by resection or laser ablation of the most active hippocampus. As an alternative, for patients with non-lesional mesial temporal epilepsy where the only question is to differentiate left versus right mesial temporal ictal onsets and neuroimaging has been unable to confirm unilateral onset, an option to replace “invasive” bitemporal EEG by diagnostic RNS could be considered to record longer, obtain more seizures and provide treatment at the same time. Ultimately, larger studies are needed to replicate these findings and create guidelines for the role and design of invasive evaluation in non-lesional temporal lobe epilepsy surgery candidates.

## CRediT authorship contribution statement

**Ghazala Perven:** Conceptualization, Methodology, Validation, Investigation, Resources, Writing - original draft, Writing - review & editing. **Irina Podkorytova:** Conceptualization, Methodology, Validation, Investigation, Resources, Writing - original draft, Writing - review & editing. **Kan Ding:** Resources, Writing - review & editing. **Mark Agostini:** Resources. **Sasha Alick:** Resources. **Rohit Das:** Resources. **Hina Dave:** Resources. **Marisara Dieppa:** Resources. **Alexander Doyle:** Resources. **Jay Harvey:** Resources. **Bradley Lega:** Resources. **Rodrigo Zepeda:** Resources. **Ryan Hays:** Conceptualization, Methodology, Validation, Investigation, Resources, Supervision, Writing - review & editing.

## Declaration of Competing Interest

The authors declare that they have no known competing financial interests or personal relationships that could have appeared to influence the work reported in this paper.
